# Model-based research toward design of innovative materials: molecular weight prediction of bridged polysilsesquioxanes†

**DOI:** 10.1039/d0ra02909b

**Published:** 2020-08-03

**Authors:** Takayoshi Ishimoto, Satoru Tsukada, Shin Wakitani, Kenji Sato, Daiki Saito, Yuki Nakanishi, Sakino Takase, Takashi Hamada, Joji Ohshita, Hiroyuki Kai

**Affiliations:** Advanced Materials Laboratory, Advanced Automotive Research Collaborative Laboratory, Graduate School of Engineering, Hiroshima University 1-4-1 Kagamiyama Higashi-Hiroshima Hiroshima 739-8527 Japan jo@hiroshima-u.ac.jp kaihi@hiroshima-u.ac.jp tishimo@yokohama-cu.ac.jp; Graduate School of Nanobioscience, Yokohama City University 22-2 Seto, Kanazawa-ku Yokohama 236-0027 Japan; Fundamentals of Model-Based Development, Graduate School of Advanced Science and Engineering, Hiroshima University 1-4-1 Kagamiyama Higashi-Hiroshima Hiroshima 739-8527 Japan; Department of Applied Chemistry, Graduate School of Advanced Science and Engineering, Hiroshima University 1-4-1 Kagamiyama Higashi-Hiroshima Hiroshima 739-8527 Japan; Division of Materials Model-Based Research, Digital Monozukuri (Manufacturing) Education and Research Center, Hiroshima University Higashi-Hiroshima 739-0046 Japan

## Abstract

Toward the design and manipulation of innovative materials, we propose a new concept called “model-based research (MBR)”. In MBR, measurable physical and chemical properties of materials are mathematically modelled by explanatory parameters obtained by computer simulation from an atomistic point of view. To demonstrate the potential of MBR, we modelled the molecular weights of a series of polysilsesquioxanes with respect to the H_2_O/silane molar ratio employed for the polymerization of monomers bis(triethoxysilyl)methane, ethane, ethylene, and acetylene (BTES-M, -E1, -E2, and -E3), as an example. The equation *y* = *ax*^*n*^ well reproduced the behaviour of the molecular weights of the BTES series, in which *a* and *n* were obtained using the calculated molecular parameters for monomers as the explanatory parameters. Detailed understanding and discussion were theoretically possible on the basis of the mathematical model. We predicted the molecular weights of polymers that would be obtained from monomers BTES-P and BTES-Ph with C_3_H_6_ and C_6_H_4_ as the spacer, respectively, using the mathematical model. Experimental validation of these polymers clearly showed the possibility of qualitative categorization. Our proposed concept, MBR, is a powerful tool to analyse materials science toward innovative materials design.

## Abstract 2

Modeling and prediction of molecular weight by MBR.

## Graphical abstract

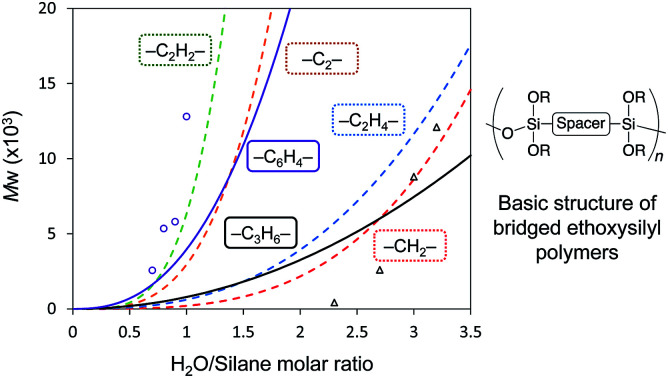

## Introduction

Materials development based on simple molecular design is generally difficult as the atomic-level molecular properties do not always relate directly to the functionalities of materials that are composed of a huge number of molecules in the condensed states. Even though the molecular properties can be predicted with high reliability by molecular simulation at a high level of theory,^1–5^ many other large-scale factors exist, including intermolecular interaction, molecular alignment, and interfacial structure, which are difficult to predict or often unpredictable yet exert a significant influence on the material functionalities. This also applies to the monomer–polymer relationship as the properties and functionalities of polymers do not always directly reflect those of monomers. For example, network or highly branched polymers have molecular weights that are difficult to control, in marked contrast to that linear polymers have molecular weights that well depend on monomer reactivity and can be much more easily controlled by, for example, the loading ratio of the monomer and the terminating and/or initiating reagent.^6–8^

**Fig. 1:**
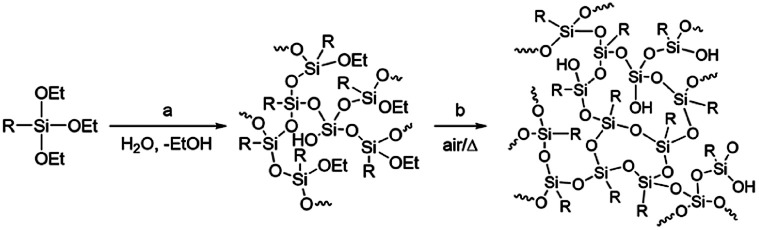
Preparation of polysilsesquioxane by hydrolysis/condensation of triethoxysilane, including controlled polymerization by partial hydrolysis/condensation of EtO–Si bonds (a) and calcination in air to further enhance network formation (b).

Polysilsesquioxanes (PSQs) have received much attention as typical organic–inorganic hybrid materials.^9–13^ They can be prepared by the hydrolysis/condensation polymerization of trifunctionalized silanes and consist of a three-dimensional siloxane network that gives rise to thermal and mechanical stability of the resulting PSQ materials. For the preparation of PSQ films, liquid PSQs prepared by controlled polymerization are coated on a substrate and then calcined at a high temperature to further facilitate the condensation, as shown in Fig. 1.^14,15^

**Fig. 2:**
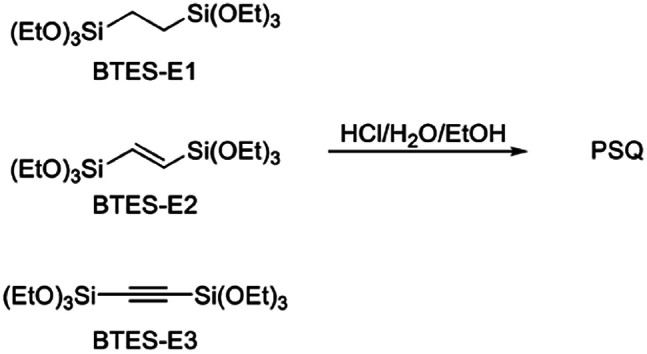
Monomers of bridged PSQs.

In this process, the control of the molecular weights of the liquid PSQs is of utmost importance. To avoid significant shrinking of the film due to condensation, which may produce cracks and/or pinholes on the film, the liquid PSQs should have as high a polymerization degree, *i.e.*, molecular weight, as possible. However, an exceedingly high polymerization degree should be avoided as it would result in the formation of non-processible solid PSQs. We were able to prepare three types of bridged PSQs from the corresponding monomers, as shown in Fig. 2.^14,15^ However, to our surprise, their polymerization behaviours were markedly different in spite of the fact that the monomer structures were nearly the same except for the bond orders of the bridging C2 units. Based on both experimental and theoretical considerations, we concluded that the different polymerization behaviours arise from the different rigidities of the C2 bridges. However, the molecular weights of such network polymers as PSQs are still difficult to predict precisely.

To approach this challenging problem, the key feature of our work is the application of machine learning^16,17^ to understand the driving force for the formation of macroscopic properties from an atomistic point of view and to predict and design new materials. The use of machine learning techniques in materials science is still in its infancy and the applications are limited to simple surface adsorption, stability of homogeneous materials, derivatives of molecules, and so on.^18–20^ To apply machine learning for macroscopic properties, such as thermal conductivity, hardness, density, and so on in complex systems, it is necessary to propose a new strategy beyond the present machine learning approaches.

In this study, we propose a new concept on the basis of machine learning toward materials design, using the control of the molecular weights of the bridged PSQs as an example. We call this “model-based research (MBR)”, which is clearly different from the conventional machine learning approach. In MBR, material properties that are usually difficult to predict are analysed by using a uniform mathematical model equation with rather accurate parameters that are readily obtained by theoretical simulation at the atomic level. Even though the relationship between the material properties and the parameters at the atomic level is not precisely explained, they should have a strong correlation that may be linked by an equation. This concept is expected to provide a new strategy for the design of materials at the atomic level.

MBR is based on the efficient automobile engine manufacturing process called “model-based development (MBD)”.^21^ The basic idea of MBD is to divide a complex whole-engine system into simple parts that can be simulated by a computer. In fact, MBD achieved a new high-performance automobile engine by the so-called “V-type development process”. Although MBD is very useful for efficient automobile engine manufacture as it reduces both cost and time, the direct application of MBD to materials development is difficult because material properties are based on a high complexity of multi-scale and multi-physics phenomena. To overcome such difficulties in materials development, we proposed the new concept, MBR, for efficient research in materials science, as mentioned above.

We believe that acquiring a fundamental understanding at the atomic level is one of the key factors for modelling material properties. To construct a model for material properties, the machine learning technique is a very powerful tool. The collaboration of three techniques, *i.e.*, measurement and analysis by experimental techniques, obtaining geometry and electronic structure by computer simulation, and modelling by machine learning, are necessary in MBR. Based on a fundamental understanding of physical and chemical properties and models, it will be possible to design and develop innovative materials. Furthermore, the constructed models may be used for the design of related materials. Here, we modelled the measured molecular weights of the bridged PSQs by using explanatory parameters based on geometry and electronic structure obtained by computer simulation as an example of MBR.

## Methods

### General procedure for synthesis

Bis(triethoxysilyl)methane (BTES-M) was purchased from Gelest, Inc. 1,4-Bis(triethoxysilyl)benzene (BTES-Ph) and chloroplatinic acid hexahydrate were purchased from Sigma-Aldrich Co. LLC. Ethanol (super dehydrated (99.5)) and 6 mol L^−1^ hydrochloric acid used as the reaction solvent and catalyst, respectively, were purchased from Wako Pure Chemical Industries. These reagents were used as received without purification. Allyltriethoxysilane and triethoxysilane were purchased from Tokyo Chemical Industry Co., Ltd. Toluene was purchased from Nacalai Tesque, Inc., distilled from calcium hydride, and dried over 4 Å molecular sieves. The structures are shown in Fig. 4. Gel permeation chromatography (GPC) was performed with a high-performance liquid chromatograph (Shimadzu) using three directly connected TSKgel G6000H columns (LC-20AD pump, CTO-20A, and an RID-10A detector (Shimadzu)) with THF as the eluent at the flow rate of 1.0 mL min^−1^. A 5–10 wt% solution of sample was introduced after filtration through a 0.20 μm diameter membrane filter (DISMIC-13JP, ADVANTEC). Polystyrene samples were used as standards. NMR spectra were measured on a Varian System500 spectrometer.

### Synthesis of 1,4-bis(triethoxysilyl)propane (BTES-P)

BTES-P was synthesized with a slight modification of a previously reported method.^22^ The reaction was carried out under dry air. A toluene solution (50 mL) of 3-(triethoxysilyl)propene (22.6 mL, 0.100 mol), triethoxysilane (22.5 mL, 0.120 mol), and chloroplatinic acid hexahydrate (62.1 mg, 0.120 mmol) was stirred for 22 hours at room temperature. Chloroplatinic acid hexahydrate (51.8 mg, 0.100 mmol) was added and the reaction mixture was stirred for another 16 hours. The reaction mixture was evaporated and distilled under reduced pressure to yield BTES-P as a clear colorless liquid (29.2 g, 79%). Bp 101–103 °C (0.21 torr). ^1^H NMR (500 MHz, CDCl_3_) *δ* 0.73 (t, ^3^*J*_HH_ = 10 Hz, 4H), 1.22 (t, ^3^*J*_HH_ = 5 Hz, 18H), 1.54–1.58 (m, 2H), 3.81 (q, ^3^*J*_HH_ = 5 Hz, 12H). ^13^C{^1^H} NMR (126 MHz, CDCl_3_) *δ* 14.46, 16.63, 18.45, 58.42. ^29^Si{^1^H} NMR (99 MHz, CDCl_3_) *δ* −45.3.

### General procedure for preparation of poly(BTES-M), poly(BTES-P), and poly(BTES-Ph)

The reactions were carried out in a fashion similar to that for the polymerization of BTES-E1, -E2, and -E3.^14,15^ Into a four-necked flask equipped with nitrogen inlet and outlet tubes and a mechanical stirrer, BTES-R (5.00 mmol) and EtOH (50.00 mmol) were charged and the reaction mixture was cooled on an ice bath for 10 minutes with stirring at 150 rpm under nitrogen flow (360 mL min^−1^). 6 mol L^−1^ Hydrochloric acid (0.525 mmol) and distilled water were added in molar ratios of 0.9–3.2 for H_2_O/BTES-monomer. The reaction mixture was stirred for an additional 10 minutes at 0 °C and then warmed to room temperature and further stirred for 10 minutes. The reaction mixture was stirred for 3 hours at 80 °C to generate the polymer as a viscous liquid. Molecular weights were determined by GPC analysis.

### Computational details

To obtain the explanatory parameters for the molecular weights of the four types of alkoxysilanes, geometry optimization calculations for the monomers were done by using B3LYP/6-31G(d) level of theory. Solvent effects from ethanol were considered by the polarized continuum model (PCM).^23^ All ethoxy groups in the monomers were substituted by hydroxyl groups in this study. As an explanatory variable for reactivity, the dimerization energy of two monomers was calculated. The proton association energy to a hydroxyl group, which is one of the most important reaction steps for dimerization, was also calculated. The flexibilities between Si and Si stretching and Si–X–Si (X = C_2_H_4_, C_2_H_2_, C_2_, CH_2_, C_3_H_6_, and C_6_H_4_) bending in the monomer structure were considered by *ab initio* molecular dynamics (MD) calculation under B3LYP/6-31G(d). The time step of 1.0 fs was used in the Verlet method.^24^ Iterative calculations were done until 10 000 steps. All electronic structure calculations were performed by using the general atomic and molecular electronic structure system (GAMESS).^25^ We also modified GAMESS program for *ab initio* MD calculations.

### Machine learning

The least absolute shrinkage and selection operator (LASSO)^26^ method was used for logistic regression analysis because this method provides a prediction model even when the number of data is smaller than that of parameters. This method can perform both variable selection and regularization to optimize the regression model while avoiding overfitting. The LASSO has been extensively used for material science fields.^27–32^ The big data analysis is generally popular approach for materials science.^27,31^ However, it is sometimes difficult to prepare many experimental or calculation data directly. Recently, the LASSO was applied to small datasets for materials science.^28^ The preparation of enough datasets for molecular weight of our target polymer is also quite difficult due to the difficulty of experiments and need long time for experimental cost. It is necessary to analyse molecular weight trend from small datasets. Therefore, the LASSO method was adopted to optimize the prediction model fitting of the experimental values with the molecular parameters. We first determined the fitting parameters of experimental results (molecular weight). The fitting parameters and calculated values from electronic structure calculations were prepared as objective and explanatory variables, respectively. By using LASSO analysis, we obtained the explanatory parameters based on electronic structure calculation for fitting parameters of experimental data of molecular weight. Detailed procedure of model equation construction in this study was shown in ESI.†

## Results and discussion

### Polymer molecular weights

**Fig. 3:**
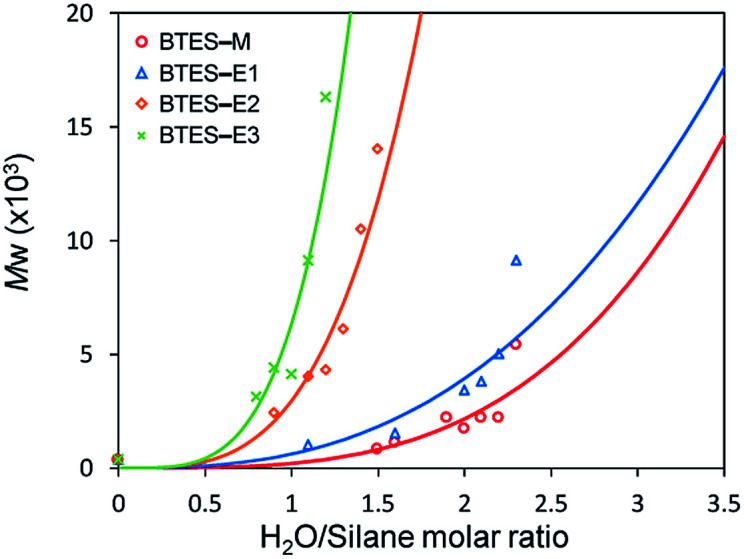
Regression curves for BTES-M, -E1, -E2, and -E3 obtained by LASSO method. Experimental results of molecular weight are also plotted.

**Fig. 4:**
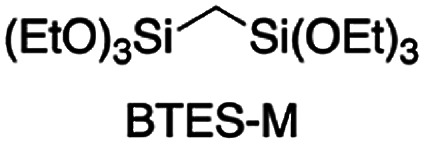
Monomer structure of BTES-M.

Fig. 3 shows the plots of molecular weight *versus* amount of water for the polymerization of BTES-E1, -E2, and -E3, as reported previously,^16^ together with those for BTES-M obtained in the present study (Fig. 4).

**Fig. 5:**
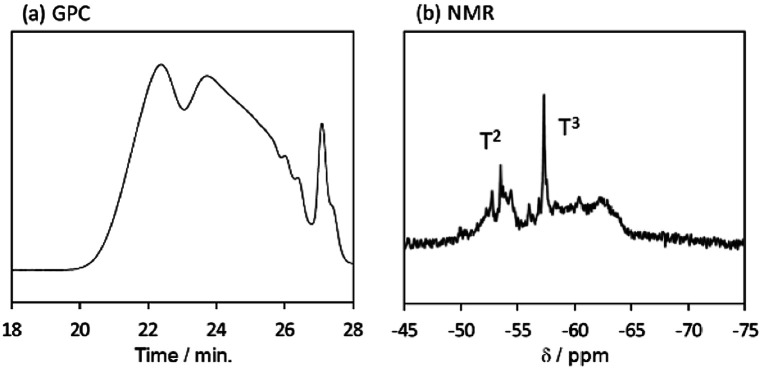
(a) GPC chart and (b) ^29^Si NMR spectrum of BTES-M. Sample was synthesized under the H_2_O/silane molar ratio = 2.6.

The behaviour of the molecular weight changes of BTES-M is similar to that of BTES-E1. To check the detailed structure of BTES-M, we analysed the gel permeation chromatography (GPC) and ^29^Si nuclear magnetic resonance (NMR) for BTES-M as shown in Fig. 5.

**Fig. 6:**
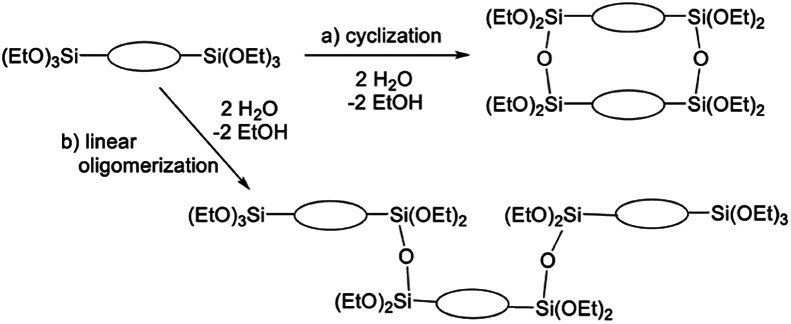
Molecular weight changes depending on the reaction modes. With two water molecules, a linear reaction gives a trimer, whereas cyclization allows only the dimer formation.

The broad multiple peaks in Fig. 5(a) indicated the growth of the polymer chain of BTES-M. A single sharp peak in the low molecular weight region means the cyclic dimer formation. In the NMR spectra shown in Fig. 5(b), the T^2^ and T^3^ mean two and three dimensional bridged structures, respectively. The T^2^ and T^3^ structures are mixed in BTES-M. The GPC chart and NMR spectrum of BTES-M were also similar to the BTES-E1.^7^ This behaviour markedly differed from our previous consideration that higher molecular weights would be obtained as molecular rigidity was enhanced. We had assumed that more flexible monomers should provide cyclic oligomers more easily, thereby suppressing the molecular weight increase (Fig. 6).

The C1 bridge of BTES-M is much less flexible than the ethylene C2 bridge of BTES-E1. However, BTES-M gave polymers with lower molecular weights than BTES-E1, indicating that there may be important factors affecting the molecular weights other than bridge flexibility.

### Fitting of molecular weight

To find a mathematical fit of the plots in Fig. 3, we assumed the equation *y* = *ax*^*n*^. Although this equation is simple, it is suitable to extract the fundamental and important factors because only two parameters are included. Based on the least squares method, the parameters in the equation, *a* and *n*, were optimized, as listed in Table 1, together with the coefficient of determination (*R*^2^). Generally, with increasing the number of parameters, the *R*^2^ is improved. While the equation *y* = *ax*^*n*^ has only two parameters, this equation well reproduced the experimental molecular weights for all the BTES series, *i.e.*, the *R*^2^ values were higher than 0.8, as shown in Table 1. Parameter *a* was the smallest for BTES-M. For the C2-linked BTES compounds, parameter *a* increased with increasing bond order of the bridges. On the other hand, parameter *n* was the smallest for BTES-E1.

**Table 1:** Optimized parameters *a* and *n* in *y* = *ax*^*n*^ and coefficients of determination of BTES-M, -E1, -E2, and -E3

	*a*	*n*	*R* ^2^
BTES-M	0.204	3.409	0.8142
BTES-E1	0.616	2.676	0.8606
BTES-E2	2.951	3.421	0.9263
BTES-E3	6.326	3.920	0.8537

### Construction of equation

To find the controlling factors for *a* and *n*, regression analysis was carried out by using the least absolute shrinkage and selection operator (LASSO) method. As the first step, we selected atomic-scale parameters that had been obtained by computer simulation of the compounds in ethanol as the potential controlling factors. Meso-scale information, such as the branching degree of the polymer structures and the reactivity of the polymers/oligomers, seems to be also important factors that control the polymerization reactions. It is also likely that larger scale information, including viscosity of the polymer solution and polymer shape (linear, coil, rod-like, *etc.*), exerts an influence on GPC analysis, thereby also affecting the molecular weights. However, these meso-scale information may be under the control of monomer molecular factors directly or indirectly. It should be worthwhile if we could find some monomer-scale parameters that strongly affect the meso-scale properties, such as the molecular weights of the polymers in the present work, as this would prove the possibility of materials design by atomic-scale tuning of geometry and electronic structure. As the explanatory parameters for objective variables *a* and *n*, geometry, electronic structure, and flexibility parameters of the monomer structures of BTES-M, -E1, -E2, and -E3 were selected. The potential explanatory parameters examined in this study are listed in Table 2.

**Table 2:** Explanatory parameters based on geometry, electronic structure, reactivity, and dynamics of unit structures of BTES-M, -E1, -E2, and -E3

Parameters	BTES-M	BTES-E1	BTES-E2	BTES-E3
Number of carbons	1	2	2	2
Number of hydrogens	2	4	2	0
H/C	2	2	1	0
Surface area (Å^2^)	162.6	195.2	188.0	181.2
Volume (Å^3^)	133.4	152.3	146.9	141.4
HOMO (Ha)	−0.2903	−0.2780	−0.2813	−0.2881
LUMO (Ha)	0.0248	0.0375	−0.0336	−0.0189
Atomic charge of Si	1.021	0.973	0.950	0.852
Atomic charge of O	−0.629	−0.625	−0.621	−0.602
Solvation energy (kcal mol^−1^)	−6.59	−12.29	−10.68	−12.70
Δ*E*_association_ (kcal mol^−1^)	5.73	4.93	3.96	−0.68
Δ*E*_dimerization_ (kcal mol^−1^)	−15.24	−5.91	−5.39	−6.34
*R*(Si⋯Si)_Ave._ (Å)	3.057	4.535	4.580	4.898
Δ*R*(Si⋯Si)^a^ (Å)	0.320	0.279	0.193	0.207
∠(Si⋯C⋯Si)_Ave._ (°)	109.7	148.0	156.1	176.7
Δ∠(Si⋯C⋯Si)^a^ (°)	16.8	15.8	9.8	11.8

a Difference of maximum and minimum values.

The numbers of carbon and hydrogen atoms in the spacers and their ratio (H/C), the surface area, and the volume of monomers are the geometry parameters of the monomers. The energy levels of the highest-occupied molecular orbital (HOMO) and the lowest-unoccupied molecular orbital (LUMO) and the atomic charges of silicon and oxygen are the electronic structure parameters. The solvation energy was also calculated and the proton association energy (Δ*E*_association_) was calculated by the following eqn (1), where X is the spacer in the unit structure (CH_2_, C_2_H_4_, C_2_H_2_, and C_2_).

1 Δ*E*_association_ = *E*((OH)_3_Si–X–Si(OH)_2_(OH_2_)^+^) − [*E*((OH)_3_Si–X–Si(OH)_3_) + *E*(H^+^)]

Proton association is considered an important step in acid-catalyzed reactions. The dimerization energy of the unit structure (Δ*E*_dimerization_) was also calculated on the basis of the following equation,

2 Δ*E*_dimerization_ = *E*((OH)_3_Si–X–Si(OH)_2_O(OH)_2_Si–X–Si(OH)_3_) + *E*(H_2_O) − 2*E*((OH)_3_Si–X–Si(OH)_3_).

To take the structural flexibility of the monomers into account, *ab initio* molecular dynamics (MD) calculations were performed for BTES-M, -E1, -E2, and -E3. We focused on the averages of the Si⋯Si distance and the Si⋯C⋯Si angle during the MD simulations. Differences between the maximum and the minimum of those motions were also analysed.

To obtain the mathematical model equation for parameters *a* and *n*, we analysed the relationship between these explanatory parameters and the objective parameters. By applying the LASSO method, which is one of the most common techniques for machine learning, three explanatory parameters were found as the important controlling factors for each of *a* and *n* in *y* = *ax*^*n*^. Based on these results, the model equations for *a* and *n* are shown in eqn (3) and (4), respectively.

3 *a* = 45.64 (atomic charge of O) − 4.04 (atomic charge of Si) − 2.00 (H/C) + 37.13

4 *n* = −2.38 (LUMO) − 0.24 (number of hydrogen atoms) − 0.01 (volume) + 5.23

The atomic charges of oxygen and silicon strongly affect parameter *a*. Parameter *n* includes a large contribution from the LUMO of the unit structure. We clearly found different controlling factors for parameters *a* and *n*. As shown in Fig. 3, these equations were clearly reproduced the experimental results. It is effective to understand the reason for the molecular weight change of PSQs and to find the key mechanisms to control the molecular weights for new targets by using these equations.

In our previous work on the reaction mechanism in the polymerization of BTES-E1, -E2, and -E3,^16^ we demonstrated that all the water used in the reactions was consumed to convert ethoxy groups into silanol or disiloxane bonds. We also found that the formation of cyclic units was mainly responsible for suppressing the molecular weight increase in BTES-E1. We, therefore, consider that the reactivity of silanol towards condensation to form disiloxane bonds and the reaction selectivity, *i.e.*, linear chain growth or cyclization, significantly affect the molecular weights of the polymers.

**Fig. 7:**
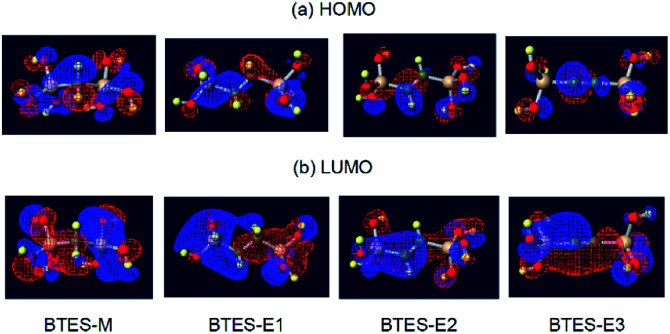
Visualization^33^ of the highest occupied molecular orbital (HOMO) and the lowest unoccupied molecular orbital (LUMO) of BTES-M, -E1, -E2, and -E3.

The atomic charges of oxygen and silicon and the H/C ratios are involved in eqn (3) as the controlling factors of *a*. The atomic charges of silicon are decreased in the order of BTES-M > BTES-E1 > BTES-E2 > BTES-E3. Silicon is a π-acceptor as well as a σ-donor.^34^ As π-acceptors, the silicon atoms in BTES-E2 and BTES-E3 accept a negative charge from ethylene and acetylene units. In fact, the σ*(Si)–π(C) interaction is clearly seen in the HOMO of these molecules in addition to the n(O)–σ*(Si) interaction (Fig. 7). For BTES-M and BTES-E1 with a saturated carbon spacer, however, the delocalization of σ- and n-electrons is observed to a smaller extent. In these molecules, the silicon atoms seem to act as a σ-donor. The smaller number of relatively more electronegative carbons in BTES-M would lead to a higher atomic charge of the silicon atoms than that in BTES-E1. Usually, the higher atomic charge of silicon and the lower atomic charge of oxygen would accelerate condensation reactions that proceed *via* a substitution reaction of oxygen nucleophile at the silicon centre. However, this contrasts with that the higher atomic charge of silicon and the lower atomic charge of oxygen decrease parameter *a*, suppressing the molecular weight increase. Therefore, it is likely that parameter *a* is related to the reaction selectivity. Entropically favoured linear chain growth seems to be relatively enhanced under the milder situation with a lower atomic charge of silicon and a higher atomic charge of oxygen, compared to cyclization. The H/C ratio reflects chain flexibility: a higher ratio leads to lower chain-growth/cyclization selectivity, thereby decreasing the molecular weight.

On the other hand, the LUMO energy level, the molecular volume, and the number of hydrogen atoms on the spacer contribute to parameter *n*. In general, the lower-lying LUMO and the higher-lying HOMO lead to higher reactivity. As can be seen in Fig. 7, the LUMOs are rather delocalized over the molecules with the contribution of silicon σ* orbitals, whereas the HOMOs are more localized mainly on the oxygen n-orbitals. As a result, the HOMO energy levels are nearly the same regardless of the monomer structure (−0.2903 eV to −0.2780 eV), whereas the LUMO energy levels widely range from 0.0375 eV to −0.0336 eV depending on the structure, thereby largely affecting parameter *n*. The lower LUMO levels for unsaturated BTES-E2 and -E3 than saturated BTES-M and -E1 are understood in terms of the σ*(Si)–π*(C) interaction. Molecular volume is related to molecular diffusion, and thus to reactivity. Increasing the number of hydrogen atoms seems to suppress reactivity due to steric hindrance of the spacer.

From the results described above, we can definitely conclude that parameters *a* and *n* have different controlling factors. Although it is not easy to explain why these explanatory parameters are selected as the controlling factors for parameters *a* and *n*, it is true that the molecular weights can be reproduced on the basis of these molecular factors of the monomers.

### Prediction of molecular weight from proposed equation

**Fig. 8:**
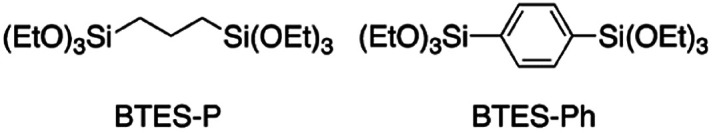
Monomer structure of BTES-P and -Mh.

Based on the proposed equation of molecular weight, the predicted curves of molecular weights were analysed. We simulated two monomers BTES-P and BTES-Ph with C_3_H_6_ and C_6_H_4_ spacers, respectively (Fig. 8).

The controlling factors for BTES-P and BTES-Ph obtained by electronic structure calculation from GAMESS are listed in Table 3.

**Table 3:** Explanatory parameters of unit structures of BTES-P and BTES-Ph

Parameters	BTES-P	BTES-Ph
Number of hydrogens	6	4
H/C	2	0.667
Volume (Å^3^)	169.3	190.6
LUMO (Ha)	0.0389	−0.0330
Atomic charge of Si	0.975	0.907
Atomic charge of O	−0.622	−0.617
*a*	0.791	3.965
*n*	2.042	2.498

**Fig. 9:**
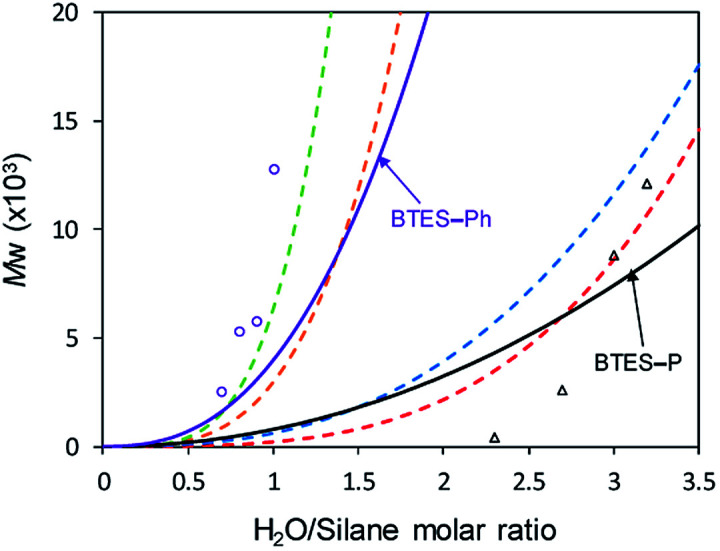
Predicted curves of molecular weights for BTES-P and BTES-Ph as shown by black and purple line, respectively. Open triangle and open circle dots are molecular weights for BTES-P and BTES-Ph, respectively, obtained by experiment. Red, blue, orange, and green dashed lines are regression curves for BTES-M, -E1, -E2, and -E3 as shown in Fig. 3.

Using those parameters, we predicted the curves of molecular weights with respect to the H_2_O/silane molar ratio, as shown in Fig. 9.

Reflecting the similarity of parameter *a*, the curve for BTES-P is close to those for BTES-M and BTES-E1, and the curve for BTES-Ph is near those for BTES-E2 and -E3. To validate this prediction, we synthesized polymers from BTES-P and BTES-Ph (Table 4) and the molecular weight changes depending on the amount of water are plotted in Fig. 9. When the ratio exceeded 1.0, gelation occurred to form insoluble polymeric materials. On the other hand, BTES-P showed no evident molecular weight changes up to water/silane ratio of 2.3 and at the ratio of 3.2 gelation occurred. The gelation of BTES-Ph at rather low H_2_O/silane ratio is likely due to the phenylene bridge that is rigid and have high tendency to form π–π stacking. Reproducibility of molecular weight determination by GPC is a little low for BTES-Ph polymers, likely due to the formation of insoluble gel to an extent, which was confirmed by Tyndall scattering of the solutions even at H_2_O/silane ratios smaller than 1.0. However, the quick molecular weight increase could be traced cleanly. Fig. 9 shows the selected plots of molecular weight changes for BTES-Ph polymerization and the all the plots are given in Fig. S2.† To compare predicted curve and experimental data of BTES-P and -Ph, two category separation which comes from difference of C–C and C=C/C≡C bonds of monomers, was succeeded. The gap between predicted curve and experimental data is improved by adding other data, although the Fig. 9 shows qualitatively good fit of the experimental plots by the predicted curves.

**Table 4:** Results of sol–gel reactions^a^

Silane	Entry	H_2_O/silane molar ratio	Molecular weight^b^
			*M*_w_/g mol^−1^
BTES-P	1	2.3	420
	2	2.7	2600
	3	3.0	8800
	4	3.2	12 100
BTES-Ph	1	0.7	2600
	2	0.8	5300
	3	0.9	5800
	4	1.0	12 800

a Scale in operation: silane 5 mmol, molar ratios: EtOH/silane = 50 mmol.

b Determined by GPC relative to polystyrene standards.

**Fig. 10:**
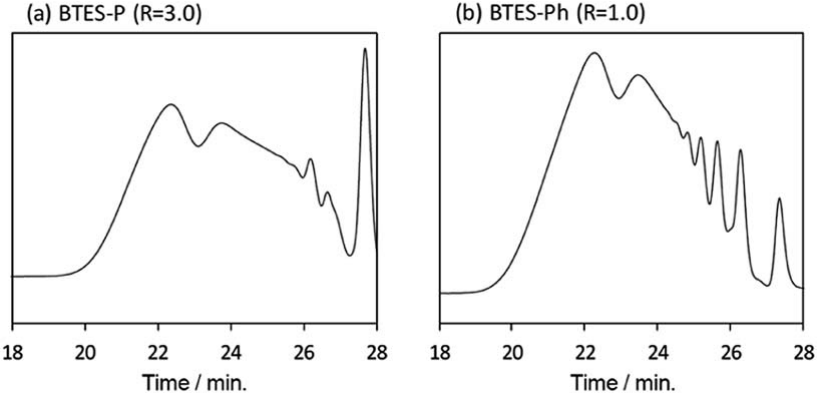
GPC charts (a) BTES-P and (b) BTES-Ph.

Compared to BTES-E1 and -M, BTES-P showed even lower molecular weights in both prediction and experiments. BTES-P is known to easily undergo intramolecular cyclization to form stable six-membered cyclic disilsesquioxanes in acid-catalysed hydrolytic polycondensation reactions.^35,36^ On the other hand, the unsaturated phenylene bridge in BTES-Ph led to the generation of high molecular weight polymers and gelation even with low water content, similar to BTES-E3. The GPC charts of BTES-P and -Ph synthesized under the H_2_O/silane ratio = 3.0 and 1.0, respectively, are shown in Fig. 10.

As well as the BTES-E1 (and -M), a single sharp peak in the low molecular weight region was observed in the case of BTES-P including single C–C single bonds. Total shape of GPC chart for BTES-Ph having linear main structure was similar to the BTES-E3 (and also -E2), although some small peaks are observed in the low molecular weight region. One of the reasons of shape difference of GPC charts is that the cyclic compounds of BTES-Ph was obtained due to the rigidity of main structure. However, overall shape of GPC chart and molecular weight curve of BTES-P and -Ph were similar to the BTES-E1, -M and BTES-E2, -E3, respectively. In this sense, we clearly demonstrated the reliable categorization of PSQ derivatives with respect to the molecular weight based on the concept of MBR.

**Fig. 11:**
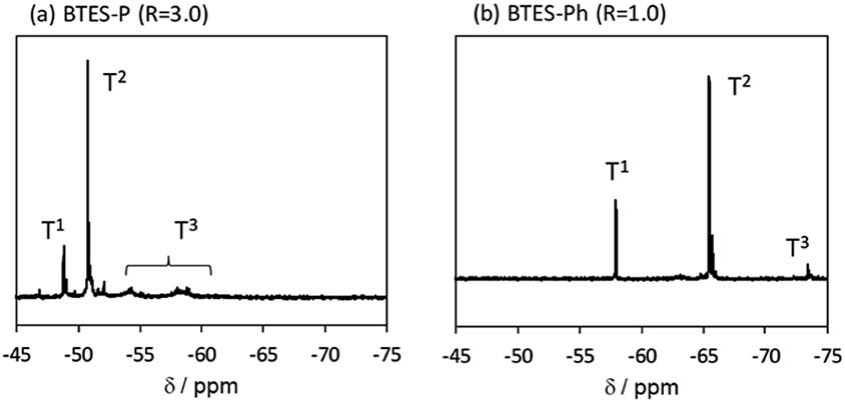
^29^Si NMR spectra of (a) BTES-P and (b) BTES-Ph.

The ^29^Si NMR spectra of BTES-P and -Ph are presented in Fig. 11, together with signal assignments based on comparison with the literature data.^16,35,36^ The spectrum of BTES-P reveals sharp T^1^ and T^2^ signals ascribed to cyclic dimers and/or cyclic units involved in the polymer structure. Relatively large and broad signals likely due to the polymer T^2^ and T^3^ structures are also seen in the spectrum, similarly to that of BTES-E1.^16^ In contrast, the spectrum of BTES-Ph shows only tiny T^3^ signals, suggesting that BTES-Ph should have more linear backbone than BTES-P, like BTES-E2 and -E3.^16^ These agree well with the categorization based on their GPC analysis mentioned above.

## Conclusions

We have proposed a new concept called “MBR”, which is based on the machine learning technique, with an eye to enhancing materials design. In MBR, macroscopic properties are mathematically modelled by explanatory parameters obtained by computer simulation from an atomistic point of view. As an example, we modelled the molecular weights of bridged PSQs by using MBR. The equation *y* = *ax*^*n*^ well reproduced the behaviour of the molecular weights of BTES-M, -E1, -E2, and -E3. Parameters *a* and *n* were modelled by using the explanatory parameters of geometry, electronic structure, reactivity, and dynamics of monomer structures. The explanatory parameters were essential to understanding the controlling factors for the polymerization of PSQs. Based on the model for molecular weight, we predicted the molecular weight theoretically. Our experimental results for the two types of spacers (C_3_H_6_ and C_6_H_4_) could be categorized on the basis of the predicted curves. This indicates that the model developed in this study includes the fundamental characteristics of molecular weight change with respect to the H_2_O/silane molar ratio, although further improvement of the model is necessary for accurate analysis. We have clearly shown the usefulness of concept of MBR for molecular weight prediction and finding of controlling factors. The MBR approach is a powerful tool in materials science with an eye to realizing innovative materials design.

## Conflicts of interest

The authors declare no competing interests.

## Supplementary Material

RA-010-D0RA02909B-s001

## References

[cit1] Zvereva E. Z., Shagidullin A. R., Katsuba S. A. (2011). J. Phys. Chem. A.

[cit2] Neese F. (2009). Coord. Chem. Rev..

[cit3] Stein T., Kronik L., Baer R. (2009). J. Am. Chem. Soc..

[cit4] Bagno A., Rastrelli F., Saielli G. (2006). Chem.–Eur. J..

[cit5] Taylor J., Guo H., Wang J. (2001). Phys. Rev. B.

[cit6] Danks A. E., Hall S. R., Schnee Z. (2016). Mater. Horiz..

[cit7] Levy D. and Zayat M., The Sol-Gel Handbook, Wiley-VHC, 2001, ch. 1

[cit8] Hench L. L., West J. K. (1990). Chem. Rev..

[cit9] Brook M. A. , Silicon in organic, organometallic, and polymer chemistry, Wiley, 2000, ch. 10

[cit10] Shea K. J., Loy D. A. (2001). Chem. Mater..

[cit11] Abe Y., Gunji T. (2004). Prog. Polym. Sci..

[cit12] Kanamori K., Nakanishi K. (2011). Chem. Soc. Rev..

[cit13] Hu L. C., Shea K. (2011). Chem. Soc. Rev..

[cit14] Abe Y., Shimano R., Arimitsu K., Gunji T. (2003). J. Polym. Sci., Part A: Polym. Chem..

[cit15] Yamamoto K., Ohshita J., Mizuno T., Tsuru T. (2014). J. Sol-Gel Sci. Technol..

[cit16] Wagner N., Rondinelli J. M. (2016). Front. Mater..

[cit17] Ramprasad R., Batra R., Pilania G., Monnodi-Kanakkitodi A., Kim C. (2017). npj Comput. Mater..

[cit18] Gasper R., Shi H., Ramasubramaniam A. (2017). J. Phys. Chem. C.

[cit19] Kim C., Pilania G., Ramprasad R. (2016). J. Phys. Chem. C.

[cit20] Yada A., Nagata K., Ando Y., Matsumura T., Ichinoseki S., Sato K. (2018). Chem. Lett..

[cit21] Wakitani S., Yamamoto T., Morishige C., Adachi T., Harada Y., Muraoka T., Niinai S. (2018). J. JSEE.

[cit22] Loy D. A., Carpenter J. P., Myers S. A., Assink R. A., Small J. H., Greaves J., Shea K. J. (1996). J. Am. Chem. Soc..

[cit23] Tomasi J., Mennucci B., Cammi R. (2005). Chem. Rev..

[cit24] Verlet L. (1968). Phys. Rev..

[cit25] Shmidt M. W., Baldridge K. K., Boatz J. A., Elbert S. T., Gordon M. S., Jensen J. H., Koseki S., Matsunaga N., Ngyuyen K. A., Su S., Windus T. L., Dupuis M., Montgomery J. A. (1993). J. Comput. Chem..

[cit26] Tibshirani K. (1996). J. R. Stat. Soc. Series B
Stat. Methodol..

[cit27] Zhow T., Song Z., Sundmacher K. (2019). Engineering.

[cit28] Zhang Y., Ling C. (2018). npj Comput. Mater..

[cit29] Ghiringhelli L. M., Vybiral J., Ahmetcik E., Ouyang R., Levchenko S. V., Draxl C., Scheffler M. (2017). New J. Phys..

[cit30] Bartók A. P., De S., Poelking C., Bernstein N., Kermode J. R., Csányi G., Ceriotti M. (2017). Sci. Adv..

[cit31] Ghiringhelli L. M., Vybiral J., Levchenko S. V., Draxl C., Scheffler M. (2015). Phys. Rev. Lett..

[cit32] Meredig B., Agrawal A., Kirklin S., Saal J. E., Doak J. W., Thompson A., Zhang K., Choudhary A., Wolverton C. (2014). Phys. Rev. B.

[cit33] Winmostar V9, X-Ability Co. Ltd., Tokyo, Japan, 2019, https://winmostar.com/

[cit34] Brook M. A. , Silicon in organic, organometallic, and polymer chemistry, Wiley, 2000, ch. 1

[cit35] Loy D. A., Carpenter J. P., Alam T. M., Shaltout R., Dorhout P. K., Small J. H., Shea K. J. (1999). J. Am. Chem. Soc..

[cit36] Loy D. A., Carpenter J. P., Myers S. A., Assink R. A., Small J. H., Greaves J., Shea K. J. (1996). J. Am. Chem. Soc..

