# Supplementary material for: Model-based research toward design of innovative materials: molecular weight prediction of bridged polysilsesquioxanes
Source: RSC Adv. 2020 Aug 3;10(48):28595–602. doi: 10.1039/d0ra02909b (PMC9055803; doi:10.1039/d0ra02909b)
Supplement: RA-010-D0RA02909B-s001 [file RA-010-D0RA02909B-s001.pdf]

Electronic Supplementary Material (ESI) for RSC Advances.
This journal is © The Royal Society of Chemistry 2020

Supplementary Information Materials

for

Model-based research toward design of innovative materials: Molecular weight prediction of bridged polysilsesquioxanes

Takayoshi Ishimoto*[a,b], Satoru Tsukada[a], Shin Wakitani[c], Kenji Sato[a], Daiki Saito[d], Yuki Nakanishi[d], Sakino Takase[d], Takashi Hamada[a,d], Joji Ohshita*[a,d,e], and Hiroyuki Kai*[a]

[a.] *Advanced Materials Laboratory, Advanced Automotive Research Collaborative Laboratory, Graduate School of Engineering, Hiroshima University, 1-4-1 Kagamiyama, Higashi-Hiroshima, Hiroshima 739-8527, Japan.*

[b.] *Graduate School of Nanobioscience, Yokohama City University, 22-2 Seto, Kanazawa-ku, Yokohama 236-0027, Japan.*

[c.] *Fundamentals of Model-Based Development, Graduate School of Advanced Science and Engineering, Hiroshima University, 1-4-1 Kagamiyama, Higashi-Hiroshima, Hiroshima 739-8527, Japan.*

[d.] *Department of Applied Chemistry, Graduate School of Advanced Science and Engineering, Hiroshima University, 1-4-1 Kagamiyama, Higashi-Hiroshima, Hiroshima 739-8527, Japan.*

[e.] *Division of Materials Model-Based Research, Digital Monozukuri (Manufacturing) Education and Research Center, Hiroshima University, Higashi-Hiroshima 739-0046, Japan.*

*Correspondence: jo@hiroshima-u.ac.jp, kaihi@hiroshima-u.ac.jp, tishimo@yokohama-cu.ac.jp

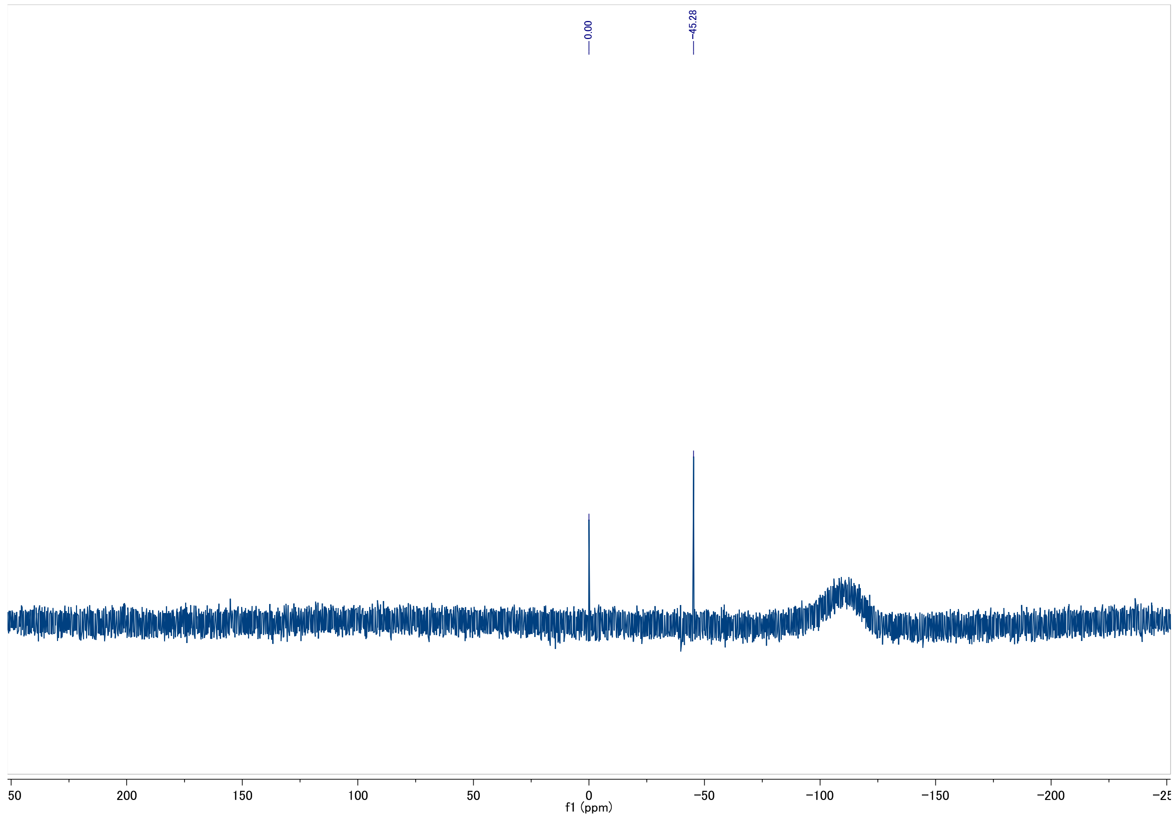

Fig. S1. $^{29}Si\{^{1}H\}$ NMR of BTES–P in $CDCl_3$.

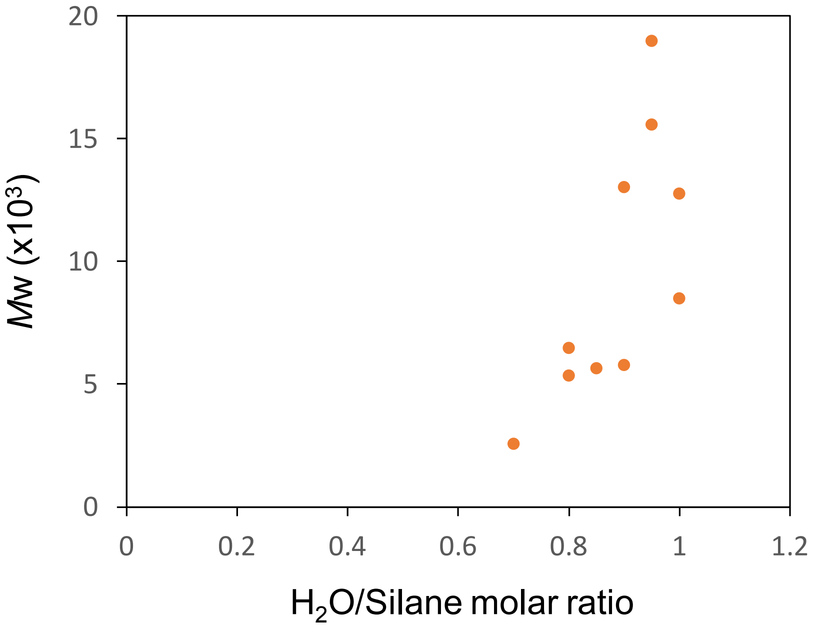

Fig. S2. Plots of GPC molecular weights (M_w) versus H_2O/silane molar ratios for polymerization of BTES–Ph.

Table S1. Results of sol-gel reactions of BTES–M, –E1, –E2, and –E3.[a]

Silane	H_2O/Silane molar ratio	Molecular weight[b] $M_w \times 10^{-3}$/g mol^{-1}	Silane	H_2O/Silane molar ratio	Molecular weight $M_w \times 10^{-3}$/g mol^{-1}
BTES–M	1.5	0.8	BTES–E2[c]	0.9	2.4
	1.6	1.1		1.1	4.0
	1.9	2.2		1.2	4.3
	2.0	1.7		1.3	6.1
	2.1	2.2		1.4	10.5
	2.2	2.2		1.5	14.0
	2.3	5.4	BTES–E3[c]	0.8	3.1
BTES–E1	1.5	0.8		0.9	4.4
	1.6	1.1		1.0	4.1
	1.9	2.2		1.1	9.1
	2.0	1.7		1.2	16.3
	2.1	2.2			
	2.2	2.2			
	2.3	5.4			

[a]Scale in operation: silane 5 mmol, molar ratios: EtOH/silane = 50 mmol.

[b]Determined by GPC relative to polystyrene standards.

[c]The Data from Ref. 1 were used.

Reference

1 K. Yamamoto, J. Ohshita, T. Mizuno, and T. Tsuru, *J. Sol-Gel Sci. Technol.*, 2014, **71**, 24-30.

Detailed procedure of model equation construction based on MBR

1. Prepare experimental data of molecular weight (BTES–M, –E1, –E2, and –E3).
2. Prepare simple equation (usually within two paprameters) for fitting of experimental data. (We also tried various types of equations having two parameters. Best values for R^2 was $y = ax^n$, in this study. a and n are objective parameters for machine learning.)
3. Calculate various electronic structure and structural values of BTES–M, –E1, –E2, and –E3 monomers by using density functional theory and molecular dynamics simulation. (16 parameters were obtained as explanatory parameters)
4. Calculate relation between objective parameters obtained from fitting of experimental data (a and n in $y = ax^n$) and explanatory parameters obtained from simulation by using LASSO.
5. Obtain model equations for a and n using selected explanatory parameters.
6. Check accuracy of model equations.
7. Analyze chemical and physical contributions to determine molecular weight.
8. Calculate explanatory parameters in model equations to obtain a and n values for unknown compounds from simulation. (in this study, BTES–P, and –Ph)
9. Draw predicted molecular weight line based on model equations for unknown compunds. (in this study, BTES–P, and –Ph)
10. Predict molecular weight line for target molecule. (In this study, we just compared and validated reliability of predicted molecular weight line for BTES–P, and –Ph)
